# F-waves persistence in peripheral sensory syndromes

**DOI:** 10.1055/s-0043-1772599

**Published:** 2023-10-04

**Authors:** Fabricio Diniz de Lima, Alberto Rolim Muro Martinez, Gabriel da Silva Schmitt, Andrea Fernandes Eloy da Costa França, Paulo Eduardo Neves Ferreira Velho, Juliana Akita, José Antônio Garbino, Anamarli Nucci, Marcondes Cavalcante França Jr

**Affiliations:** 1 Universidade Estadual de Campinas, Faculdade de Ciências Médicas, Departamento de Neurologia, Campinas SP, Brazil.; 2 Hospital Alemão Oswaldo Cruz, Setor de Neurofisiologia Clínica, São Paulo SP, Brazil.; 3 Universidade Estadual de Campinas, Faculdade de Ciências Médicas, Departamento de Clínica Médica, Campinas SP, Brazil.; 4 Instituto Lauro de Souza Lima, Setor de Neurofisiologia Clínica, Bauru SP, Brazil.

**Keywords:** Nerve Conduction Studies, Polyneuropathies, Mononeuropathies, Hereditary Sensory and Autonomic Neuropathies, Estudos de Condução Nervosa, Polineuropatias, Mononeuropatias, Neuropatias Hereditárias Sensoriais e Autônomas

## Abstract

**Background** The distinction between sensory neuronopathies (SN), which is by definition purely sensory, and sensory polyneuropathies (SP) and sensory multineuropathies (SM) is important for etiologic investigation and prognosis estimation. However, this task is often challenging in clinical practice. We hypothesize that F-wave assessment might be helpful, since it is able to detect subtle signs of motor involvement, which are found in SP and SM, but not in SN.

**Objective** The aim of the present study was to determine whether F-waves are useful to distinguish SN from SP and SM.

**Methods** We selected 21 patients with SP (12 diabetes mellitus, 4 transthyretin familial amyloid polyneuropathy, 4 others), 22 with SM (22 leprosy), and 26 with SN (13 immune-mediated, 10 idiopathic, 3 others) according to clinical-electrophysiological-etiological criteria. For every subject, we collected data on height and performed 20 supramaximal distal stimuli in median, ulnar, peroneal, and tibial nerves, bilaterally, to record F-waves. Latencies (minimum and mean) and persistences were compared across groups using the Kruskal-Wallis and Bonferroni tests. *P* -values < 0.05 were considered significant.

**Results** All groups were age, gender, and height-matched. Overall, there were no significant between-group differences regarding F-wave latencies. In contrast, F-wave persistence was able to stratify the groups. Peroneal F-wave persistence was higher, bilaterally, in the SN group compared to SM and SP ( *p* < 0.05). In addition, F-waves persistence of the ulnar and tibial nerves was also helpful to separate SN from SP ( *p* < 0.05).

**Conclusion** F-wave persistence of the peroneal nerves might be an additional and useful diagnostic tool to differentiate peripheral sensory syndromes.

## Resumo

**Antecedentes** A distinção entre neuronopatias sensitivas (SN) e polineuropatias sensitivas (SP) e multineuropatias sensitivas (SM) é importante para a investigação etiológica e para o prognóstico. Contudo, esta tarefa é desafiadora na prática clínica. Hipotetizou-se que a avaliação das ondas-F pode ser útil, por ser capaz de detectar envolvimento motor nas SP e SM, mas não nas SN.

**Objetivo** Determinar se as ondas-F podem ajudar a distinguir entre SN, SP e SM.

**Métodos** Selecionou-se 21 pacientes com SP (12 diabetes mellitus, 4 ATTR-FAP e 4 com outras neuropatias), 22 com SM (22 hanseníases) e 26 com SN (13 imunomediadas, 10 idiopáticas e 3 com outras neuronopatias), de acordo com critérios clínicos, etiológicos e eletrofisiológicos. Para cada indivíduo, foi aferida a altura e foram aplicados 20 estímulos distais supramáximos nos nervos mediano, ulnar, fibular e tibial, bilateralmente, para registrar as ondas-F. Uma comparação foi feita, por grupo, das latências (mínimas e médias) e persistências pelos testes Kruskal-Wallis e Bonferroni. Valores de *p* < 0.05 foram considerados estatisticamente significativos.

**Resultados** Todos os grupos foram pareados por idade, sexo e altura. Não houve diferença estatística significativa entre os grupos quanto às latências das ondas-F. A persistência da onda-F foi capaz de estratificar os grupos, sendo as dos nervos fibulares bilateralmente maiores no grupo SN que nos grupos SM e SP ( *p* < 0.05). Adicionalmente, a persistência das ondas-F dos nervos ulnares e tibiais também foi útil para distinguir SN de SP ( *p* < 0.05).

**Conclusão** A persistência das ondas-F dos nervos fibulares pode ser uma ferramenta adicional e útil para diferenciar síndromes sensitivas periféricas.

## INTRODUCTION

Peripheral neuropathies with predominant or almost exclusive sensory involvement are frequent in clinical practice. These peripheral sensory syndromes can be divided into three major groups: sensory polyneuropathies (SP), sensory multineuropathies (SM), and sensory neuronopathies (SN). Sensory polyneuropathies typically present with length-dependent and symmetrical deficits. They are often found in association with systemic or genetic conditions, such as diabetes or transthyretin familial amyloid polyneuropathy (ATTR-FAP). 1 In contrast, SM are characterized by multifocal involvement of sensory nerves, that is, a sensory mononeuropathy multiplex. Patients have asymmetrical areas of hypoesthesia and arms are frequently more affected than legs. A remarkable example of SM is leprosy-related neuropathy, endemic in certain areas of the world. 2 Finally, the target of damage in SN is the dorsal root ganglia leading to sensory ataxia and asymmetric sensory deficits. 3 However, SN may also present with diffuse and rather symmetric deficits depending on the disease duration and the underlying etiology (genetic subtypes of SN, such as Friedreich ataxia, Machado-Joseph disease and *RFC1* -related disorders, are often symmetric indeed). 4 5 6

The distinction between these three conditions is relevant for the practicing neurologist, because the etiological work-up for each syndrome is obviously different. In particular, SN should be clearly recognized, since recent neurophysiological data suggest that the therapeutic window is rather short for this subgroup of diseases. 7 Nerve conduction study (NCS) and electromyography (EMG) are the cornerstone for the differential diagnosis, but it is sometimes challenging for the clinical neurophysiologist. For instance, there are two peculiar scenarios in which it seems to be harder to distinguish SP from SN: patients with mild asymmetries or doubtful length-dependent pattern. Hence, different strategies have been investigated to distinguish between SN and SP, 8 9 10 some of which offer promising results, like the ulnar sensory-motor amplitude ratio (USMAR) and the sural/radial amplitude ratio (SRAR). 8 11 12 However, few studies included subjects with SM. This is a rarer sensory syndrome, especially in Europe/USA, but its clinical phenotype is the one with highest overlap with SN.

In the present study, we looked at F-waves as an additional tool to assist in the differential diagnosis between SN and SM/SP. These are late responses obtained after supramaximal stimulation of peripheral nerves. 13 14 They assess not only distal, but also proximal portions of motor axons. For that reason, F-waves are significantly more sensitive than standard conduction studies to identify subtle motor involvement in peripheral neuropathies. 15 16 17 In this scenario, we hypothesized that F-waves would be able to help distinguish SN, which are, by definition, purely sensory, from SM and SP. These last two syndromes almost always present motor damage, even though the latter is sometimes missed in routine NCS/EMG. To accomplish this goal, we recruited a cohort of patients with SN, SP, and SM to undergo detailed F-wave studies of upper and lower limb nerves. We then compared different F-wave parameters in the three groups looking at the potential usefulness to separate them.

## METHODS

### Study design and subjects' selection

This was a unicenter, observational, analytical, and prospective study performed at Universidade de Campinas (UNICAMP) in Brazil.

We recruited adult patients between 18 and 80 years old with exclusive sensory complaints due to a peripheral nerve disease, regularly followed at the UNICAMP neuromuscular outpatient clinic from March 2017 to August 2018. We then classified each patient into one of the three peripheral sensory syndromes based exclusively on neurological examination, routine NCS/EMG, and laboratorial investigation:

- SP: a known etiology that commonly leads to sensory polyneuropathy (e.g. diabetes mellitus and transthyretin familial amyloid polyneuropathy) and symmetrical, length-dependent findings considering both clinical and electrophysiological abnormalities;
- SM: patients with asymmetric and/or non-length-dependent electrophysiological findings in at least one nerve, absence of sensory ataxia, and confirmed leprosy diagnosis;
- SN: patients with non-length-dependent sensory abnormalities and relatively preserved motor findings on electrophysiological evaluation, fulfilling the clinical and electrophysiological criteria of Camdessanché et al. 18

**Figure 1:**
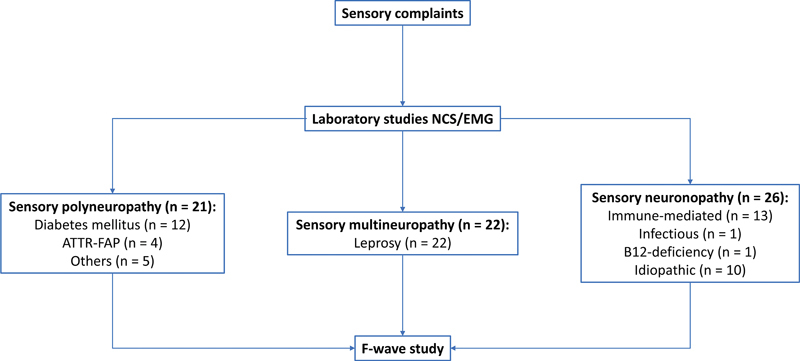
Flowchart summarizing the design of the study and the etiological profile of patients in each group that underwent F-wave evaluation. NCS/EMG: nerve conduction study and electromyography.

After this three-group categorization, in order to assess the F-wave role in the group discrimination, we especially subjects to undergo further neurophysiological testing (described below) within a period of up to 6 months. Figure 1 displays a flowchart summarizing the design of the study and the etiological profile of the three groups.

This study complied with the Declaration of Helsinki and was approved by our Institutional Review Board (CAAE 25789013.5.0000.5404). All individuals signed an informed consent before any study-related procedure.

### Clinical criteria and evaluation

Patients were considered with peripheral nervous syndrome (PNS) when their complaints included negative clinical sign or symptom (such as sensory loss to pain and temperature, sensory loss to vibration and proprioception, ataxia, clumsiness, areflexia) or/and positive clinical sign or symptom (pain and dysesthesias). Moreover, these complaints were put into context of a PNS taking into account neurological examination, correlation with symptom time course, and identification of known risk factors. Patients with clinical signs and/or subsequent electrodiagnosis suggestive of radiculopathy were excluded.

### Neurophysiologic evaluation

Nerve conduction studies and electromyography were performed in patients using the Neuropack M1 MEB-9200J electromyographer (Nihon Kohden Corp., Shinjuku City, Tokyo, Japan). All procedures were performed at a standardized skin temperature of the examined limb (32–34 °C) and took place in a quiet and temperature-controlled room (23–26° C).

We reviewed available NCS/EMG data prior to enrollment to check whether patients met the inclusion criteria. These included sensory and motor nerve conduction studies according to the standard protocol of our lab. Sensory nerve action potentials (SNAPs) were recorded through antidromic techniques described elsewhere, using standardized distances for electric stimuli and between electrodes 19 in the median, ulnar, radial, and sural nerves (reference values for amplitude and velocity: > 20 µV/50 m/s, > 17 µV/> 50 m/s, > 15 µV/> 50 m/s and > 6 µV/> 40 m/s, respectively). Compound muscle action potentials (CMAPs) were recorded orthodromically in the median, ulnar, tibial, and peroneal nerves (reference values for amplitude base to peak and velocity: > 6 mV/> 50 m/s, >6 mV/> 50 m/s, >4 mV/> 40 m/s and > 2.5 mV/> 40 m/s, respectively). Proximal entrapment neuropathies (particularly for ulnar and peroneal nerves) were ruled out by performing NCS across the elbow and the fibular head, as described elsewhere. 19

For all recruited patients, we recorded the F-waves by a standardized protocol: 20 supramaximal stimuli in the distal site of routine conduction motor nerves, frequency set at 0.5 Hz, stimulator placed with the anode more distal and cathode more proximal, gain in 200 μV, 10 ms sweep and low- and high-frequency filters set at 2 Hz and 10 kHz, respectively. The minimum response amplitude considered to determine the F-waves was 20 μV peak to peak. 20 We assessed the following F-wave properties: minimal and mean latencies and the persistence (defined as the percentage of stimuli capable of obtaining a F-wave). This evaluation was performed by a neurophysiologist blinded to the etiological diagnosis.

### Statistical analysis

The Kolmogorov-Smirnov test was applied and determined a non-normal distribution ( *p* < 0.05) for neurophysiological data. Clinical, demographic, and basic NCS data were shown through descriptive statistics. F-wave latencies (minimum and mean) and persistence were compared using the Kruskal-Wallis test followed by a posthoc Bonferroni analysis. For those parameters able to discriminate the groups, we plotted receiver operator characteristic (ROC) curves to measure the area under the curve (AUC) and assess diagnostic accuracy. Bonferroni-corrected *p* -values < 0.05 were considered significant.

## RESULTS

### Demographics, clinical, and nerve conduction data

Table 1 summarizes the demographic and clinical data of all patients. There were no significant differences across the groups regarding age, gender, and height. Diabetes and ATTR-FAP were the most frequent causes for SP, whereas all patients with SM had leprosy. Regarding the SN group, most patients had underlying immune-mediated mechanisms: Sjögren syndrome (n = 4), autoimmune hepatitis (n = 3), systemic lupus erythematosus (n = 1), and FGFR3-related (n = 5). One patient with SN had a toxic etiology: chemotherapy-induced by oxaliplatin and another one an infectious etiology: human T-cell lymphotropic virus type I (HTLV-1). 21 Even though HTLV-1 could cause damage to other PNS sites (e. g., anterior horns), this last patient was extensively investigated along 15 years of follow-up and found to have no additional involvement beyond the dorsal root ganglia.

**Table 1:** Demographic and clinical data of all patients included in the study

		SP (n = 21)	SM (n = 22)	SN (n = 26)	Group comparison (Kruskal Wallis *p* -value)
Demographic data	Age, years old - median value (IQR)	56 (20)	54 (23)	52 (17)	0.512
	Height, cm - median value (IQR)	168 (8)	165 (14.5)	163 (12.5)	0.390
	Sex (F:M)	8:13	9:13	16:10	0.209
Clinical data	Sensory ataxia	10	9	24	**< 0.001** ^▲●^
	Paresthesia/hypoesthesia	19	8	20	**< 0.001** ^▪●^
	Neuropathic pain	13	19	16	0.120

Abbreviations: IQR, interquartile range; SM, sensory multineuropathy; SN, sensory neuronopathy; SP, sensory polyneuropathy.

Notes: ^▪^ SP x SM; ^▲^ SP x SN; ^●^ SM x SN. Results are stratified for each diagnostic group.

Routine nerve conduction data ( Table 2 ) revealed a pattern of purely sensory involvement in all groups with remarkable reduction of SNAP amplitudes but preserved nerve conduction velocities. Altogether these findings suggest an axonal pattern of involvement of sensory fibers. Sensory nerve action potential abnormalities were symmetric and restricted to the lower limbs in the SP group, but it was asymmetric in the remaining groups (most noticeable in the sural and ulnar nerves). A distinctive aspect of the SN group was the extensive abnormality in upper limb SNAPs. It is noteworthy that 11 patients with SP had prolonged distal motor latencies for the median nerve (ranging from 4.0–8.6 ms), suggestive of concomitant carpal tunnel syndrome (CTS). The distribution of abnormal amplitudes on sensory nerve conduction studies is shown in Table 3 .

**Table 2:** Routine nerve conduction data of all patients included in the study

Nerve conduction study Median value (IQR)		SP (n = 21)	SM (n = 22)	SN (n = 26)	Group comparison (Kruskal Wallis *p* -value)
Motor nerves					
Median (left / right)	Amplitude (mV)	6.9 (1.3) / 7.9 (2.5)	8.2 (4.9) / 9.3 (4.0)	10.0 (4.0) / 9.0 (4.2)	**0.005**^▲^ / **0.005** ^▲^
	NCV (m/s)	50.1 (6.4) / 49.0 (5.4)	55.7 (5.5) / 54.5 (6.6)	54.3 (6.2) / 53.0 (4.8)	**0.005**^▪^ / **0.001** ^▪▲^
Ulnar (left / right)	Amplitude (mV)	7.1 (2.4)/ 7.7 (3.4)	7.6 (3.8) / 9.1 (4.2)	8.5 (3.3) / 8.4 (2.7)	0.065 / 0.055
	NCV (m/s)	51.8 (11.7) / 52.2 (8.2)	56.5 (8.2) / 59.3 (6.7)	56.8 (4.8) / 57.6 (9.0)	0.064 / **0.004** ^▪▲^
Peroneal (left / right)	Amplitude (mV)	1.4 (4.2) / 1.5 (2.7)	3.9 (4.0) / 3.0 (3.9)	3.8 (2.7) / 4.1 (2.9)	**0.014**^▪▲^ / **0.003** ^▲^
	NCV (m/s)	38.9 (11.0) / 40.3 (6.5)	44.0 (6.2) / 43.6 (5.8)	43.6 (4.9) / 42.7 (3.2)	0.067 / **0.032** ^▪^
Tibial (left / right)	Amplitude (mV)	2.9 (6.8) / 3.2 (5.0)	7.7 (5.2) / 6.9 (6.2)	9.7 (5.7) / 10.8 (4.6)	**0.002**^▲^ / **0.001** ^▲^
	NCV (m/s)	40.0 (7.5) / 40.5 (6.1)	42.9 (6.9) / 42.4 (6.4)	42.1 (4.9) / 41.9 (4.3)	**0.017**^▪▲^ / 0.151
Sensory nerves					
Median (left / right)	Amplitude (µV)	11.5 (13.9) / 9.7 (10.4)	22.2 (21.1) / 21.0 (23.8)	2.9 (6.6) / 2.7 (6.8)	**0.000**^▲●^ / **0.000** ^▲●^
	NCV (m/s)	49.1 (12.9) / 47.4 (19.9)	50.7 (9.7) / 50.2 (8.6)	51.0 (8.1) / 53.3 (8.4)	0.141 / 0.063
Ulnar (left / right)	Amplitude (µV)	11.8 (12.5) / 9.7 (10.0)	15.1 (19.4) / 17.2 (24.4)	0.0 (5.8) / 0.0 (3.0)	**0.000**^▲●^ / **0.000** ^▲●^
	NCV (m/s)	49.6 (6.4) / 48.5 (7.8)	50.0 (7.7) / 51.5 (6.8)	44.0 (8.7) / 53.4 (12.2)	0.865 / 0.594
Radial (left / right)	Amplitude (µV)	16.0 (11.3) / 10.5 (12.8)	16.9 (18.8) / 12.0 (15.5)	0.0 (4.0) / 0.0 (5.0)	**0.000**^▲●^ / **0.001** ^▲●^
	NCV (m/s)	52.0 (11.0) / 52.5 (12.8)	54.3 (6.9) / 53.8 (4.5)	51.9 (4.1) / 54.8 (8.7)	0.687 / 0.942
Superficial peroneal (left/right)	Amplitude (µV)	0.0 (3.3) / 0.0 (3.1)	4.7 (10.4) / 2.2 (7.6)	0.0 (0.0) / 0.0 (0.0)	**0.040**^●^ / 0.120
	NCV (m/s)	40.0 (3.6) / 41.6 (1.3)	45.5 (5.0) / 44.7 (9.3)	57.7 (2.7) / 62.0 (5.0)	**0.018**^▲^ / **0.013** ^▲●^
Sural (left / right)	Amplitude (µV)	0.5 (5.2) / 0.0 (2.2)	3.3 (7.9) / 4.6 (9.7)	2.3 (5.7) / 0.0 (2.7)	0.556 / 0.144
	NCV (m/s)	44.5 (7.1) / 43.3 (4.7)	44.5 (6.2) / 46.2 (6.3)	48.6 (15.6) / 44.1 (7.2)	0.633 / 0.677

Abbreviations: IQR, interquartile range; NCV, nerve conduction velocity; SM, sensory multineuropathy; SN, sensory neuronopathy; SP, sensory polyneuropathy.

Notes: ^▪^ SP x SM; ^▲^ SP x SN; ^●^ SM x SN. Results are stratified for each diagnostic group.

**Table 3:** Distribution of abnormal amplitudes on sensory nerve conduction studies

	SP (n = 21)	SM (n = 22)	SN (n = 26)	Group comparison (Kruskal Wallis *p* -value)
Median, (left / right)	74% / 84%	45% / 45%	100% / 100%	**< 0.001** ^▲●^ **/< 0.001** ^▲●^
Ulnar, (left / right)	68% / 74%	55% / 50%	100% / 100%	**0.001** ^▲●^ **/< 0.001** ^▲●^
Radial, (left / right)	42% / 61%	55% / 55%	100% / 96%	**< 0.001** ^▲●^ **/ 0.003** ^▲●^
Superficial peroneal, (left / right)	82% / 89%	57% / 76%	88% / 82%	0.065 / 0.636
Sural, (left / right)	79% / 84%	59% / 59%	78% / 91%	0.263 / **0.026** ^●^

Abbreviations: SM sensory multineuropathy; SN sensory neuronopathy; SP, sensory polyneuropathy.

Notes ^: ▪^ SP x SM; ^▲^ SP x SN; ^●^ SM x SN.

#### F-wave analyses

**Figure 2:**
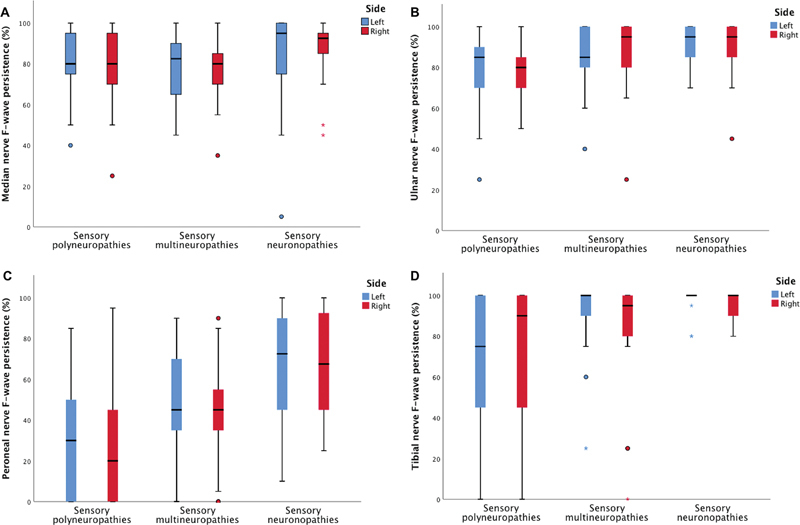
Boxplots showing the distribution of F-wave persistence for the median ( **A** ), ulnar ( **B** ), peroneal ( **C** ) and tibial ( **D** ) nerves in left (blue boxes) and right (red boxes) sides stratified for the three groups.

Detailed results of the F-wave studies for all nerves are shown in Table 4 . We failed to identify significant changes between the groups in terms of F-wave latencies. The only exception was the left median nerve that had prolonged minimal and mean latencies in the SP compared to the SM ( *p* = 0.008 and 0.027, respectively) and SN ( *p* = 0.021 and 0.049, respectively) groups; nonetheless, when excluding patients with CTS from the SP group, we did not detect any significant differences neither in minimal ( *p* = 0.816 between-group) nor in mean latencies ( *p* = 0.930 between-group). Considering the persistence, we found normal mean values in all nerves considering the 3 groups and significantly higher values for peroneal nerves on both sides in the SN when compared to the SP and SM groups ( Figure 2 ). Indeed, the persistence in ulnar and tibial nerves also helped to separate SN and SP groups ( *p* = 0.033 and 0.007, ulnar left and right and *p* < 0.001 and *p* = 0.012, tibial left and right).

**Table 4:** F-wave latencies and persistences for all nerves tested

F-wave parameter Median value (IQR)		SP (n = 21)	SM (n = 22)	SN (n = 26)	Group comparison (Kruskal Wallis *p* -value)
Median (left / right)	Minimum latencies (ms)	29.5 (5.5) / 29.9 (5.5)	26.7 (3.9) / 26.5 (2.9)	27.2 (3.2) / 26.7 (6.1)	**0.027**▪ / 0.035
	Mean latencies (ms)	31.1 (4.4) / 31.9 (4.4)	29.4 (2.9) / 29.2 (3.5)	29.3 (3.7) / 29.0 (3.7)	**0.035**▪ / 0.045
	Persistence (%)	80.0 (20.0) / 80.0 (25.0)	82.5 (23.7) / 80.0 (13.7)	95.0 (25.0) / 92.5 (10.0)	0.157 / 0.095
Ulnar (left / right)	Minimum latencies (ms)	29.8 (4.2) / 30.6 (6.1)	27.4 (4.6) / 27.5 (5.3)	27.8 (4.1) / 27.7 (3.9)	0.062 / 0.133
	Mean latencies (ms)	31.6 (3.7) / 32.2 (5.6)	29.7 (3.8) / 29.0 (4.6)	30.4 (3.8) / 30.0 (3.7)	0.287 / 0.112
	Persistence (%)	85.0 (20.0) / 80.0 (15.0)	85.0 (18.7) / 95.0 (16.2)	95.0 (13.7) / 95.0 (15.0)	**0.033**^▲^ / **0.007** ^▲^
Peroneal (left / right)	Minimum latencies (ms)	50.8 (56.9) / 46.1 (56.2)	45.9 (9.7) / 47.3 (11.0)	48.4 (6.0) / 46.4 (9.9)	0.684 / 0.916
	Mean latencies (ms)	53.4 (61.4) / 48.9 (59.2)	48.5 (11.9) / 49.3 (15.0)	53.4 (3.8) / 50.9 (10.7)	0.331 / 0.579
	Persistence (%)	30.0 (50.0) / 20.0 (45.0)	45.0 (33.7) / 45.0 (17.5)	72.5 (42.5) / 67.5 (43.7)	**0.001**^▲●^ / **0.000** ^▲●^
Tibial (left / right)	Minimum latencies (ms)	51.5 (10.6) / 50.8 (11.6)	47.4 (9.1) / 48.9 (5.3)	51.1 (6.1) / 51.5 (6.7)	0.372 / 0.625
	Mean latencies (ms)	58.9 (11.9) / 56.1 (10.8)	52.8 (9.4) / 52.8 (6.6)	54.8 (4.5) / 54.9 (5.1)	0.304 / 0.482
	Persistence (%)	75.0 (55.0) / 90.0 (55.0)	100 (10.0) / 95.0 (18.7)	100 (0.0) / 100 (10.0)	**0.000**^▲^ / **0.012** ^▲^

Abbreviations: IQR interquartile range; SM sensory multineuropathy; SN sensory neuronopathy; SP, sensory polyneuropathy.

Notes: ^▪^ SP x SM, ^▲^ SP x SN, ^●^ SM x SN. Results are stratified for each diagnostic group.

**Figure 3:**
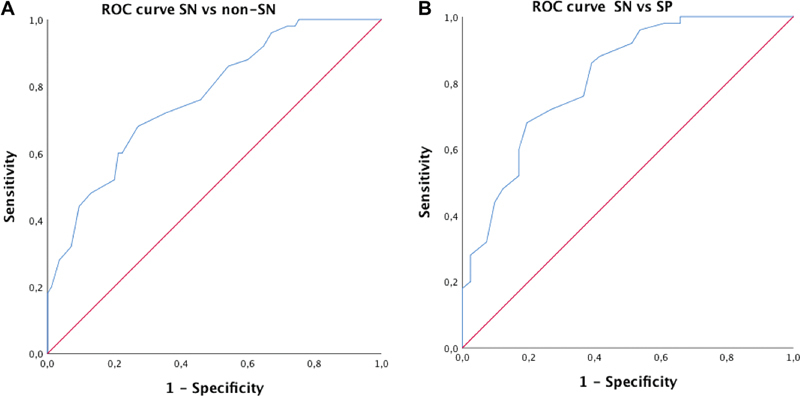
Receiver operator characteristics curves showing the diagnostic accuracy of peroneal nerve F-wave persistence to differentiate sensory neuronopathies from non-sensory neuronopathies ( **A** ) and sensory neuronopathies from sensory polyneuropathies ( **B** ).

Since the peroneal nerves proved useful to stratify the three groups, we opted to compute and plot ROC curves to assess the diagnostic accuracy ( Table 5 ). Diagnostic accuracy was reasonable for the distinction between SN and non-SN patients (AUC = 0.77) and notably between SN and SP (AUC = 0.82) ( Figure 3 and Table 5 ), especially for peroneal nerves. For comparison purposes, we looked at the diagnostic usefulness of USMAR and SRAR to distinguish between SN and non-SN. ulnar sensory-motor amplitude ratio on both sides was able to distinguish SN from non-SN ( *p* < 0.001 bilaterally). In contrast, SRAR was not capable of distinguishing between these syndromes in our series.

**Table 5:** Diagnostic accuracy of peroneal nerve F-wave persistence for the differential diagnosis of peripheral sensory syndromes

	SN vs non-SN			SN vs SP		
	Peroneal nerve	Tibial nerve	Ulnar nerve	Peroneal nerve	Tibial nerve	Ulnar nerve
Sensitivity	68%	80%	71%	68%	80%	71%
Specificity	73%	57%	54%	80%	62%	67%
AUC	0.77	0.71	0.66	0.82	0.76	0.74
Threshold	52.5%	97.5%	87.5%	52.5%	97.5%	87.5%

Abbreviations: AUC, area under the curve; SM, sensory multineuropathy; SN, sensory neuronopathy; SP, sensory polyneuropathy.

## DISCUSSION

Previous studies have already explored tibial H-reflex in the differential diagnosis of SN and SP. 9 Here, we explored the potential usefulness of another late response—the F-wave—in a similar scenario. Our primary goal was to check if parameters derived from F-wave recordings would be able to separate SN from the other sensory syndromes. To tackle this question, we took into account some methodological aspects. In contrast to previous studies, we compared SN not only with typical SP, but also with SM. 22 This is important because SM and SN often have similar standard nerve conduction findings, which turns the recognition into a challenge for clinical neurophysiologists. Moreover, leprosy—by far, the most frequent cause of SM—is endemic and clinically relevant in many parts of the world. 23 Each group—SP, SM, and SN—was also defined according to strict criteria that included clinical and NCS/EMG data as well as syndrome-specific etiologies (e.g., diabetes/ATTR-FAP for SP, leprosy for SM, Sjögren's syndrome for SN). Using this design, we showed that F-wave parameters of the ulnar, tibial, and, especially, peroneal nerves were able to distinguish SN from SM and SP. Diagnostic accuracy was reasonable for the distinction between SN and non-SN patients (AUC = 0.77) and, notably, between SN and SP (AUC = 0.82) ( Figure 3 and Table 5 ). In our series, USMAR, but not SRAR, was able to distinguish SN from non-SN. In a previous study that assessed USMAR in differential diagnosis between SN and SP, the diagnostic accuracy was slightly higher (AUC = 0.929). However, we must consider that only patients with SP were included in this last study and all of them had a single etiology (diabetes). The accuracy of USMAR still needs to be validated in the SM vs SN comparison.

F-wave persistence of the peroneal nerve was the most useful parameter to distinguish between groups. It is a measure of the excitability of the pool of α-motor neurons evaluated and is not affected by age or height. 14 20 We found that the persistence of F-waves showed normal mean values in all nerves considering the three groups; however, it was higher in the SN group compared to the SM and SP groups. In accordance with ROC curve analyses, peroneal nerve persistence was the most useful from a clinical point of view ( Table 5 ). A threshold of 52.5% for this nerve could distinguish SN from non-SN patients with a sensitivity and a specificity of 68% and 73%, respectively. The diagnostic yield for the SN x SP distinction was even higher, with a threshold of 52.5%, sensitivity of 68%, and specificity of 80%. In general, subjects with SN had remarkably high F-wave persistence values—even for the peroneal nerve, whose persistence is usually lower 24 —and this was most noticeable in the regions with more intense sensory deficits and/or sensory nerve conduction abnormalities. Considering all three peripheral sensory syndromes, only SN causes relevant sensory deafferentation at the anterior horns (lesion in SP or SM is distal to the dorsal root ganglia and only in SN proximal), that is, a disconnection between sensory (posterior horn) and motor (anterior horn) synapses. 25 Taken all these data into account, our findings suggest that such loss of afferent inputs may increase the excitability of α-motor neurons. This in line with animal data, in which dorsal rhizotomy resulted in dramatic structural and electrochemical motoneuronal changes mainly due to the loss of excitatory glutamatergic synapses. 26 Additional indirect supporting evidence for this hypothesis comes from a previous study. 27 These authors found that vigorous stimulation of the sensory nerves (through transcutaneous electrical nerve stimulation) resulted in profound inhibition of F-wave responses in both normal and spastic subjects.

The left minimal and mean F-wave latencies of the median nerves were prolonged in the SP group compared to the SN and SM groups. Such finding was probably due to the high frequency of carpal tunnel syndrome (11/21) in this subgroup as observed in the analysis after excluding patients with CTS. One must remember that the two most frequent etiologies for SP were diabetes and ATTR-FAP, which are independently associated with higher risk for CTS. 28 29 None of the remaining F-wave latency measurements in any of the remaining nerves was able to stratify the three groups. Since all three groups were age and height-matched, one cannot attribute the negative results to these confounding variables. Despite the different lesion topographies within the PNS, all three groups—SP, SM, and SN—had axonal substrate. So, it is not surprising that the latencies were similar across groups. We believe that this parameter would be more useful in the distinction between SN and demyelinating peripheral sensory syndromes, such as sensory chronic inflammatory demyelinating polyneuropathy or chronic immune sensory polyradiculpathy. 30 31 Additional studies should include this subtype of neuropathy to further validate the usefulness of F-waves in the work up of sensory neuropathies in general.

The assessment of F-waves was the focus of the current study, but some noteworthy findings were noticed for other neurophysiological parameters. Motor nerve conduction velocities in the arms were indeed significantly lower in SP and SM compared with SN. This indicates that subtle motor NC signs can be detected in the former two groups, even when clinical presentation is purely sensory. In contrast, both clinical and NCS motor function are essentially preserved in subjects with SN because damage is confined to the dorsal root ganglia. Another potential explanation for slowing of motor nerve conduction velocity (NCV) regards the etiologies of SP and SN. Many of them are associated with myelin compromise, either by direct damage (in case of leprosy or diabetes) or by focal entrapments (in case of ATTR-FAP and diabetes).

In conclusion, we have shown that F-wave persistence of the peroneal nerves may help to distinguish SN from SP and SM. This is particularly noteworthy because F-wave studies of peroneal nerves are not routinely performed in many EMG labs. In addition, F-waves persistence of the ulnar and tibial nerves was also helpful to separate SN from SP. In the evaluation of patients with a peripheral sensory syndrome but doubtful length-dependency and/or asymmetry, high F-wave persistence gives a diagnostic clue favoring SN. Since this is an easy, tolerable, and non-time-consuming technique, we advocate its general use in this diagnostic setting following the protocol herein reported.
